# Adaptation of the Quality Indicator for Rehabilitative Care (QuIRC) for use in mental health supported accommodation services (QuIRC-SA)

**DOI:** 10.1186/s12888-016-0799-4

**Published:** 2016-04-14

**Authors:** Helen Killaspy, Sarah White, Sarah Dowling, Joanna Krotofil, Peter McPherson, Sima Sandhu, Maurice Arbuthnott, Sarah Curtis, Gerard Leavey, Stefan Priebe, Geoff Shepherd, Michael King

**Affiliations:** Division of Psychiatry, University College London, Maple House, 149 Tottenham Court Road, London, W1T 7NF UK; Population Health Research Institute, St George’s, University of London, Cranmer Terrace, London, SW17 0RE UK; Unit for Social and Community Psychiatry, Newham Centre for Mental Health, Queen Mary University of London, London, E13 8SP UK; North London Service User Research Forum, Division of Psychiatry, University College London, Maple House, 149 Tottenham Court Road, London, W1T 7NF UK; Department of Geography, Durham University, South Rd, Durham, DH1 3LE UK; Bamford Centre for Mental Health & Wellbeing, University of Ulster, Northland Road, Derry Londonderry, Northern Ireland BT48 7JL UK; Centre for Mental Health, Maya House, 134-138 Borough High St, London, SE1 1LB UK

**Keywords:** Mental health, Supported accommodation, Quality assessment, Standardised tool

## Abstract

**Background:**

No standardised tools for assessing the quality of specialist mental health supported accommodation services exist. To address this, we adapted the Quality Indicator for Rehabilitative care-QuIRC-that was originally developed to assess the quality of longer term inpatient and community based mental health facilities. The QuIRC, which is completed by the service manager and gives ratings of seven domains of care, has good psychometric properties.

**Methods:**

Focus groups with staff of the three main types of supported accommodation in the UK (residential care, supported housing and floating outreach services) were carried out to identify potential amendments to the QuIRC. Additional advice was gained from consultation with three expert panels, two of which comprised service users with lived experience of mental health and supported accommodation services. The amended QuIRC (QuIRC-SA) was piloted with a manager of each of the three service types. Item response variance, inter-rater reliability and internal consistency were assessed in a random sample of 52 services. Factorial structure and discriminant validity were assessed in a larger random sample of 87 services.

**Results:**

The QuIRC-SA comprised 143 items of which only 18 items showed a narrow range of response and five items had poor inter-rater reliability. The tool showed good discriminant validity, with supported housing services generally scoring higher than the other two types of supported accommodation on most domains. Exploratory factor analysis showed that the QuIRC-SA items loaded onto the domains to which they had been allocated.

**Conclusions:**

The QuIRC-SA is the first standardised tool for quality assessment of specialist mental health supported accommodation services. Its psychometric properties mean that it has potential for use in research as well as audit and quality improvement programmes. A web based application is being developed to make it more accessible which will produce a printable report for the service manager about the performance of their service, comparison data for similar services and suggestions on how to improve service quality.

**Electronic supplementary material:**

The online version of this article (doi:10.1186/s12888-016-0799-4) contains supplementary material, which is available to authorized users.

## Background

Mental health rehabilitation services focus on people with severe and complex problems, many of whom have a diagnosis of psychosis with associated ‘negative’ symptoms that impair their motivation and organisational skills to manage everyday activities and put them at risk of self-neglect [1–3]. This group often require lengthy treatment and graduated support from specialist inpatient and community based rehabilitation services to facilitate their recovery and successful community living. Mental health supported accommodation services are a key component of this “whole system” mental health rehabilitation care pathway [4]. Although there is a lack of clarity in the published literature about exactly what is meant by the term “supported accommodation”, in the UK there are three main types; residential care, supported housing and floating outreach [5]. Residential care homes are communal facilities, staffed 24 h a day, where day to day necessities such as meals, supervision of medication and cleaning are provided to, on average, 15–20 residents. Individuals can be supported to gain skills for more independent living but these placements are not usually time-limited. Supported housing is usually provided in shared or individual tenancies with staff based on-site up to 24 h a day. These tend to be time limited placements with an average of 10–15 residents and a focus on rehabilitation, with the expectation that the person will be supported to gain skills to move on to a more independent tenancy. Floating outreach services provide support to an average of 30 people living in independent, time-unlimited tenancies. Staff are based off-site and visit each client a number of times each week to assist them with practical issues and provide emotional support, with the expectation that the amount of support can be gradually reduced and eventually stopped. In the UK, individuals will often move through this pathway, graduating from a placement with higher to lesser support every few years as their skills and confidence improve, with the ultimate aim of successfully managing an independent tenancy without the need for floating outreach support.

Although only around 10 % of people newly diagnosed with psychosis develop the kinds of complex needs that require mental health rehabilitation and supported accommodation services [6] this “low volume, high needs” group absorbs around 50 % of the total mental health and social care budget [7]. In 2006 it was estimated that around 12,500 people with mental health problems in England were living in a nursing or residential care home [8] and around 24,000 people were receiving a specialist mental health floating outreach service [9]. The number living in supported housing has not been estimated nationally. Despite the high level of resource required by this group, there have been few studies assessing the effectiveness of mental health supported accommodation services [10, 11]. Little is known about the type and quality of the support provided or how best to target this support to achieve optimal outcomes.

To address this, we are carrying out a national programme of research into mental health supported accommodation in England, the QuEST study (Quality and Effectiveness of Supported Tenancies for people with mental health problems) funded from 2012 to 2017 by the National Institute of Health Research (http://www.ucl.ac.uk/quest). The programme comprises a number of separate, related work packages: adaptation of an existing quality assessment tool for mental health supported accommodation; a national survey of mental health supported accommodation in England; a qualitative investigation of staff and service user experiences of mental health supported accommodation; a cohort study investigating outcomes for users of mental health supported accommodation services over 30 months; and a feasibility trial comparing the effectiveness of two existing models of mental health supported accommodation-supported housing and floating outreach. The first three work packages of the QuEST study received approval from the Harrow Research Ethics Committee (reference 12/LO/2009).

This paper reports on the first work package (WP1), the adaptation of an existing quality assessment tool (the Quality Indicator for Rehabilitative Care, QuIRC) for mental health supported accommodation services. The QuIRC is an international, standardised tool that assesses quality of care in longer term inpatient and community based communal mental health facilities for people with complex needs. It was developed though a pan-European study involving ten countries [12]. Its content was derived from a systematic literature review of the components of care provided in such settings [13], Delphi exercises with service users, practitioners, carers and advocates from each country [14] and a review of relevant care standards in each country. It is completed by the service manager and provides descriptive data and quality ratings of seven domains of care (Living Environment; Therapeutic Environment; Treatments and Interventions; Self-management and Autonomy; Social Interface; Human Rights; Recovery Based Practice). It has excellent inter-rater reliability [15] and good correlation with standardised measures of service users’ autonomy and experiences of care [16]. Thus, it can provide a proxy-assessment of service users’ views of a facility even though it is completed by the unit manager. It is available as a web based resource (www.quirc.eu) and takes around 45 min to complete.

## Methods

The content of the QuIRC was first reviewed by the research team to identify irrelevant or inappropriately phrased items. Three staff focus groups were recruited from North London, one each from the three main types of supported accommodation in England (residential care, supported housing and floating outreach), to gain participants’ views on the relevance of individual QuIRC items. Services where the Chief Investigator (HK) already had good links were selected for potential participation and an initial letter explaining the purpose of the study and inviting their participation was sent to the service managers. The researchers (JK, PMcP and SS) then contacted service managers to arrange a time to meet to discuss the study in more depth. Where service managers were willing, a further meeting with staff was then arranged to explain the purpose of the study. Finally, a date for a focus group was arranged. All participants in each focus group received a participant information sheet about the study and had at least 2 days to read it and address queries to the researchers before giving their informed consent to participate.

Prior to the focus groups, all participants were sent a copy of the QuIRC to review. Participants were asked to note any issues relating to appropriateness of individuals items for their setting. All focus groups were facilitated by one researcher, with another researcher taking notes. All sessions were also recorded. At the commencement of the focus group, the facilitator gave a broad description of the study, an overview of the QuIRC and the purpose of the focus group. The facilitator then led the participants through the QuIRC, eliciting comments and suggestions relating to items potentially requiring amendment. Focus group participants were also prompted to provide general comments about the structure, terminology and content of the tool. All comments were noted by the second researcher.

After the completion of each focus group, recordings were transcribed in full by the researchers. The transcription and the notes taken at the focus groups were then reviewed and a summary document prepared, listing all participant suggestions and comments.

The data from the focus groups were supplemented by the advice of three panels of experts who also reviewed the QuIRC. The first panel comprised five members with expertise in supported accommodation (two senior clinicians, a service manager, a senior policy advisor, and a senior mental health adviser to the UK’s registration body for healthcare facilities, the Care Quality Commission). The second expert panel was the QuEST study service user reference group which comprises three members with lived experience of specialist mental health supported accommodation and services. The third expert panel was the North London Service User Research Forum which comprises 12 members with lived experience of mental health problems and expertise in mental health services research. All three expert panels were sent the original QuIRC and a document summarising the comments from the focus groups. They were asked about the suggested amendments and any additional items. The first expert panel sent their comments by email. Face to face meetings were arranged with the other two expert panels to gain their feedback, attended by HK, SD, GL and the researchers. The researchers collated all comments from the focus groups and expert panels, identifying where there was consensus for adaptation, deletion or addition of a new item. These were reviewed on an item by item basis by the QuEST Programme Management Group (comprising HK and all co-investigators on the QuEST study and attended by the researchers and programme manager) to gain final agreement on changes (see Additional file 1: Appendix 1 for specific details). The adapted QuIRC was then piloted with three service managers (one from each of the three types of supported accommodation) in North London and the Programme Management Group agreed final amendments to wording in response to this.

Supported accommodation services were selected randomly for inter-rater reliability testing of the revised QuIRC. These services were selected from all supported accommodation services (residential care, supported housing and floating outreach) in each of 14 nationally representative Local Authority areas of England. These 14 areas were selected using the same sampling strategy developed by SP in a previous telephone survey of mental health supported accommodation in England, where each area was rated on an index which took account of local mental health morbidity, social deprivation, degree of urbanisation, provision of community mental health care, provision of residential care, mental health care spend, and housing demand [17]. The researchers first contacted key Local Authority personnel in each of the 14 areas to gain details of all local residential care, supported housing and floating outreach services in each area. Services were then grouped by service type and area, and randomised within each group using the RAND function in Microsoft Excel. The researchers aimed to recruit two services from each service type/area group, with the aim of recruiting 20 managers from each type of service, a total of 60 services being adequate to assess inter-rater reliability and internal consistency of the adapted QuIRC. The researchers contacted service managers to gain their informed consent for participation. Each manager received an information sheet about the study and had an opportunity to ask any questions about its purpose and process before giving written informed consent. Two researchers then interviewed the participating service managers; whilst one researcher led the interview, asking the manager to answer each adapted QuIRC item in turn, both researchers rated the adapted QuIRC independently. Where two services could not be recruited from a service type/area group, additional services from the same group, with index scores closest to the service that had not been recruited, were approached for potential participation. This occurred in ten areas for residential care, six areas for supported housing and eight areas for floating outreach.

### Data analysis

Data were entered by the researchers into an SPSS database developed by the study statistician (SW). Analysis of the spread of response to individual items and inter-rater reliability of the adapted QuIRC was carried out by SW. Items were considered to have inadequate response spread if > 90 % of service managers gave the same response. Internal consistency of domain scores was assessed using Cronbach’s alpha. A Cronbach’s alpha above 0.7 is considered acceptable when assessing internal consistency [18]. Inter-rater reliability was assessed using Kappa coefficients for categorical data (weighted Kappa if > 2 categories) and intraclass correlation coefficients (ICC) for normally distributed, continuous data; a Kappa coefficient [19] or ICC [20] of > 0.8 and 0.75 respectively is considered excellent agreement.

Response variance and internal consistency of the adapted QuIRC were subsequently reassessed using a larger sample of services (*n* = 87) participating in a national survey of supported accommodation services (a separate component of the QuEST study - WP2). A small number of these services participated in both WP1 and WP2. The Kaiser-Meyer-Olkin (KMO) [21] statistic is a measure of sampling adequacy (the proportion of variance among the variables that might be common variance). A KMO value of at least 0.5 is considered acceptable. The KMO statistic was assessed for the larger WP2 sample.

An exploratory factor analysis was also carried out using the larger WP2 sample to assess whether items loaded onto the adapted QuIRC domains to which they had been allocated during the development of the QuIRC and its subsequent revision. This replicated the approach taken in the original development of the QuIRC [13], where items were considered to load onto a factor (domain) if the item had a loading > ±0.3. All analyses were conducted using IBM SPSS Statistics v21 for Windows [22].

## Results

Each staff focus group comprised four members including service managers and support workers. A total of 28 QuIRC items were rephrased, 20 items were deleted and 10 items were added. The final version had 143 full items (with some items having sub-sectons). It was agreed that since floating outreach services are not “building based” but provide visiting support to people living in an independent tenancy, the items relating to the Living Environment of the service were not relevant and therefore the adapted QuIRC would not be able to provide a rating on this domain for these services. Inter-rater reliability of the QuIRC was carried out with managers of 14 residential care homes, 21 supported housing and 17 floating outreach services. The lower recruitment in residential care was due to a smaller sampling pool, whereas in floating outreach, fewer service managers responded to the invitation to participate. Table 1 shows the items with narrow response spread across the initial 52 services that took part in WP1 and the larger WP2 sample of 87 services. Of the 143 adapted QuIRC items, only 16 had a narrow range of response in the WP1 sample. Two of these widened when retested with the larger WP2 sample, but four further items showed a low response spread with the larger sample.

**Table 1:**
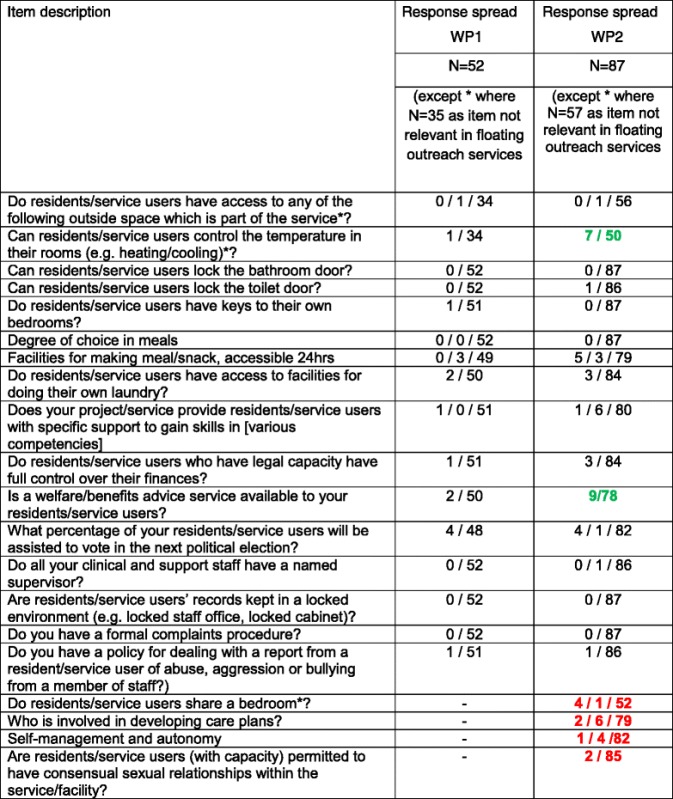
Adapted QuIRC items with low response variance

indicates response spread increased with larger WP2 sample (<90 % respondents rated item the same)

indicates response spread reduced with larger WP2 sample (>90 % respondents rated the item the same)

Tables 2 and 3 shows the adapted QuIRC domain scores and internal consistency for the initial sample (WP1) and larger sample (WP2). Internal consistency was inadequate for the Living Environment, Self-management and Autonomy, Social Integration and Human Rights domains (Cronbach’s alpha < 0.6) with the WP1 sample. It increased with the WP2 sample but remained below 0.7. The KMO statistic for all domains was greater than 0.5 when the larger sample was tested.

**Table 2:** Adapted QuIRC domain scores and internal consistency

WP1 data domain	Number of items scoring per domain	Number of services where data available	Mean (SD) score	Min-Max	Internal consistency (Cronbach’s alpha)
Living Environment	20	35	81.0 (7.1)	62.3–94.3	0.39
Therapeutic Environment	33	52	62.2 (7.3)	48.5–78.9	0.66
Treatments and Interventions	27	52	55.1 (8.4)	36.7–76.3	0.66
Self-management and Autonomy	33	52	69.0 (5.8)	53.7–81.8	0.40
Social Interface	7	52	59.0 (10.8)	33.9–89.7	0.27
Human Rights	21	52	86.7 (5.0)	71.4–96.7	0.09
Recovery-based practice	18	52	71.7 (8.2)	51.9–91.4	0.53

**Table 3:** Adapted QuIRC domain scores, internal consistency and sampling variance (KMO statistic)

WP2 data domain	Number of items scoring per domain	Number of services where data available	Mean (SD) score	Min-Max	Internal consistency (Cronbach’salpha)	KMO
Living Environment	19	57	81.2 (8.7)	53.9–96.2	0.56	0.58
Therapeutic Environment	33	87	61.4 (6.9)	38.2–75.4	0.66	0.51
Treatments and Interventions	27	87	54.2 (8.1)	35.1–73.2	0.64	0.61
Self-management and Autonomy	33	87	68.0 (6.9)	39.3–83.8	0.62	0.58
Social interface	7	87	58.9 (12.1)	37.6–85.6	0.49	0.56
Human Rights	21	87	85.5 (6.9)	66.1–97.5	0.37	0.53
Recovery-based practice	18	87	69.2 (9.9)	31.8–90.5	0.67	0.57

Table 4 shows the difference in adapted QuIRC domain scores between the three different types of supported accommodation for the WP1 and WP2 samples.

**Table 4:** Difference in adapted QuIRC domain scores across service types[values are mean (sd) min-max]

WP1 data domain	Residential care (*n* = 14)	Supported housing (*n* = 21)	Floating outreach (*n* = 17)	F (*p*-value)
Living Environment	79.0 (7.5) 62.26–88.68	82.3 (6.7) 71.70–94.34	N/A	1.9 (0.180)
Therapeutic Environment	62.0 (8.5) 49.07–78.57	65.3 (6.3) 51.80–78.89	58.7 (6.1) 48.54–67.21	4.3 (0.020)
Treatments and Interventions	60.1 (8.8) 47.72–75.72	56.3 (8.4) 36.65–76.26	49.5 (4.2) 41.34–56.05	8.3 (0.001)
Self-management and Autonomy	69.7 (5.6) 60.51–78.67	70.4 (6.4) 53.69–81.81	66.8 (4.6) 59.14–74.38	2.0 (0.150)
Social Interface	59.1 (12.7) 40.69–89.74	60.6 (10.9) 33.89–80.17	56.9 (9.2) 37.42–69.02	0.5 (0.587)
Human Rights	82.8 (5.1) 71.38–91.77	84.9 (5.4) 74.45–95.65	87.1 (4.6) 78.65–95.01	2.2 (0.128)
Recovery-based practice	68.6 (11.4) 51.93–88.86	75.4 (6.2) 64.58–91.42	69.5 (5.6) 56.62–76.63	4.2 (0.021)
WP2 data domain	Residential care (*n* = 22)	Supported housing (*n* = 35)	Floating outreach (*n* = 30)	F (*p*-value)
Living Environment	78.3 (10.0) 53.9–96.2	83.0 (7.2) 61.5–96.1	NA	4.2 (0.045)
Therapeutic Environment	58.1 (7.8) 38.2–71.6	65.4 (5.4) 55.1–75.4	59.2 (5.6) 47.8–71.1	12.7 (<0.001)
Treatments and Interventions	54.1 (6.8) 38.7–63.0	58.9 (7.1) 45.2–73.2	48.8 (6.9) 35.1–66.0	17.2 (<0.001)
Self-management and Autonomy	64.6 (8.7) 39.3–78.2	71.7 (5.6) 57.9–83.8	66.2 (4.7) 58.0–75.1	10.6 (<0.001)
Social Interface	54.1 (8.9) 39.7–67.3	68.2 (10.4) 42.2–85.6	51.7 (8.4) 37.6–67.4	29.0 (<0.001)
Human Rights	79.5 (7.8) 66.1–96.5	85.9 (5.3) 74.3–97.5	89.6 (4.5) 77.3–97.3	19.5 (<0.001)
Recovery-based practice	63.4 (11.8) 31.8–86.2	75.5 (7.2) 57.8–90.5	66.2 (6.6) 50.2–77.5	16.8 (<0.001)

The supplementary Table (Additional file 2: Appendix 1) shows the results of the inter-rater reliability testing of the adapted QuIRC. A total of 70 ICC analyses were conducted and only one item was found to be unreliable (ICC < 0.75). A total of 186 Kappa coefficient analyses were conducted and ten component parts from five items were found to be unreliable (Kappa < 0.8). In addition, there were 14 items where analyses could not be conducted due to too few cases (five items), zero variance (two items) or where variables were constants (seven items).

Exploratory factor analysis was conducted in order to establish that all items allocated to each adapted QuIRC domain loaded onto that domain. Items with zero variance were removed before this analysis, namely Living Environment, 2 items; Self-management and Autonomy, 3 items; Human Rights, 4 items. All domains had a KMO statistic > 0.5 and all items loaded onto a factor within that domain at the > ±0.3 level.

## Discussion

We adapted a quality assessment tool that had been developed for longer term mental health units, for use in mental health supported accommodation services. Amendments were made on the basis of suggestions provided by staff focus groups and feedback from expert panels, including service user groups. The adapted QuIRC comprised 143 items of which only 18 showed a narrow range of response. Inter-rater reliability was excellent, with only six items being found to be unreliable. The adapted QuIRC domain scores from the larger WP2 sample were found to differ significantly between the types of service, supporting its discriminant validity; supported housing services generally scored higher than the other two types of supported accommodation. Our exploratory factor analysis showed that the adapted QuIRC items loaded onto the domains to which they had been allocated, supporting its content validity. However, internal consistency was inadequate. Although our sampling variance (KMO statistic) gave us confidence that our domains were sufficiently coherent, the estimates of internal consistency (Cronbach’s alpha) may have been limited by the small sample size. We recruited slightly fewer services in the first phase of the programme than planned and although the internal consistency increased with the larger sample of services recruited in the second phase, the number of services tested was smaller than desirable for robust estimates of psychometric properties (generally 300 observations) which may explain this finding. An alternative explanation is that although items were grouped into coherent, logical domains, individual items within these were not designed to assess exactly the same construct but to collect information about specific, aspects of care which may or may not be correlated statistically. In other words, internal consistency may not be as relevant for this kind of tool as it would be for, say, a psychological test.

The Programme Management Group agreed amendments to the QuIRC in response to these results. The revised tool was named the Quality Indicator for Rehabilitative Care-Supported Accommodation version (QuIRC-SA). Additional explanatory information was added to improve the reliability of one item, one item was dropped completely and three items that had unreliable response options were dropped.

It was agreed that items with inadequate variance should be kept as to drop them would disrupt the logical flow of the QuIRC-SA and greater variance would be likely to be achieved in future development of the tool for settings outside the UK.

We are developing a web based version of the QuIRC-SA, just as was done with the original QuIRC. This will increase its accessibility and will mean less time to complete it than in a face to face interview. The web based application will produce a printable report for the service manager about the quality of their service on the QuIRC-SA domains, comparison data for similar services and suggestions for how to improve quality.

We tested external validity of the original QuIRC which showed good correlation with standardised measures of service users’ autonomy and experiences of care [16]. Further assessment of the QuIRC-SA’s test-retest reliability and external validity will be needed to have full confidence in its use as a tool for research and quality improvement.

## Conclusions

We adapted an existing quality assessment tool developed for longer term mental health facilities (the QuIRC) for use in supported accommodation services. The adapted tool (QuIRC-SA) has acceptable item response spread, inter-rater reliability and discriminant validity. Internal consistency of each domain was inadequate but is likely to improve when tested on a larger sample and may not be critical for this kind of measure. Exploratory factor analysis confirmed the validity of item allocation to domains. An on-line format of the QuIRC-SA is being developed in order for supported accommodation service managers to monitor the quality of their services directly. The tool has potential for use in audit, research and quality improvement programmes in this area. For example, the QuIRC has been used in national programmes of research into mental health rehabilitation services in the UK and Portugal and it has been incorporated into the Royal College of Psychiatrists’ Centre for Quality Improvement peer accreditation scheme for inpatient mental health rehabilitation services. The QuIRC-SA could have similar applications.

### Availability of data and materials

All data supporting our findings will be shared on request.

### Consent for publication

Not applicable.

### Ethics approval and consent to participate

The first three work packages of the QuEST study received approval from the Harrow Research Ethics Committee (reference 12/LO/2009). All focus group participants provided written informed consent to take part including their willingness for anonymised findings to be used in publications and reports.

## Additional files

Additional file 1: Appendix 1 gives details of the specific amendments made to the QuIRC items following the staff focus groups and expert panel reviews. (DOCX 33 kb) Additional file 2: Table S1 contains results of the item level inter-rater reliability analyses of the adapted QuIRC. (DOCX 62 kb)

## Abbreviations

ICC: intraclass correlation coefficient
KMO: Kaiser-Meyer-Olkin statistic
QuEST: quality and effectiveness of supported tenancies for people with mental health problems
QuIRC: quality indicator for rehabilitative care
QuIRC-SA: quality indicator for rehabilitative care-supported accommodation version
SPSS: statistical package for the social sciences
UK: United Kingdom
WP1: work package 1
WP2: work package 2
