# Supplementary material for: Adaptation of the Quality Indicator for Rehabilitative Care (QuIRC) for use in mental health supported accommodation services (QuIRC-SA)
Source: BMC Psychiatry. 2016 Apr 14;16:101. doi: 10.1186/s12888-016-0799-4 (PMC4831104; doi:10.1186/s12888-016-0799-4)
Supplement: Additional file 1: — Appendix 1 gives details of the specific amendments made to the QuIRC items following the staff focus groups and expert panel reviews. (DOCX 33 kb) [file 12888_2016_799_MOESM1_ESM.docx]

**Appendix 1. QuIRC item amendments agreed following focus group and expert panel review**

| **Action** | **Old**  **Item #** | **Original Wording** | **Summary of focus group comments** | **New**  **Item #** | **New Wording** |
| --- | --- | --- | --- | --- | --- |
| Amendment | N/A | Patients/residents | The term “patient” is inappropriate to setting. Replacement suggestions: “service user”, “resident”, “client” or “customer”. | N/A | Residents/service users |
| Amendment | N/A | “Unit” and “Facility” | Terms are inappropriate to setting. Replacement suggestions: “service” or “project”. | N/A | Project/service |
| Amendment | 6 | How many staff, including visiting staff, work in your unit? | Term ‘visiting staff’ is unclear; item can be interpreted to mean bank staff from within organization, external specialists, care coordinators (from CMHTs) maintenance staff etc. Terminology should be clarified. | 6 | How many support/clinical staff work in your project/service? |
| Amendment | 7 | Which of the following types of staff, including visiting staff, either work in your unit or can be accessed outside of the unit by your patients/residents?   - Psychiatrist - Clinical psychologist - Occupational therapist - Nurse - Unqualified support worker - Social worker - Counsellor/ psychotherapist - Volunteer - Arts therapist - Other | Item does not reflect frequency of contact or intensity of input from identified staff. Change “unqualified support worker” to “support worker” or “support officer”. Consider adding “welfare benefits adviser”, “employment and education adviser”, “probation officer”, “police”, “safeguarding officer” , “housing officer”, “life skills worker”, “advocate (MCA)” and “cook”. | 7 | Which of the following types of staff either work in your project/service or visit from outside the project/service?   - Psychiatrist - Clinical psychologist - Occupational therapist - Nurse - Support worker - Social worker - Counsellor/ psychotherapist - Volunteer - Arts therapist (art, dance, music, drama) - Drug and alcohol worker - Other |
| Amendment | 8 | How many unit and visiting staff are in substantive posts (i.e. not temporary or locum staff)? | Suggested by PMG | 8 | How many staff are in substantive posts (i.e. not temporary or locum staff)? |
| Amendment | 9 | What level of support does your unit provide to patients/residents? (please tick one box):   - 24 hour staff who are based at the unit and awake at night - 24 hour staff who are based at the unit and asleep at night - less than 24 hour on-site support (If less than 24 hour on-site support, please describe) | The existing answer options are too general and not exhaustive. More answer options should be added to differentiate between high, medium and low support settings. Add a blank space for managers to specify how many hours of support are offered at each level. | 9 | What level of support does your project/service provide to residents/service users? (please tick one box):   - 24 hour nursing/residential care home - 24 hour supported tenancies (waking night staff) - 24 hour supported tenancies (sleeping night staff) - Less than 24 hour supported tenancies (staff on site) - Less than 24 hour supported tenancies (staff off site) - Floating outreach (permanent independent tenancy supported by floating outreach staff) - If floating outreach, approximately how many hours contact do staff make, on average with each service user per week? - Other (please describe) |
| Amendment | 11 | Is there a maximum length of stay? | Term “maximum” is misleading; most services have move-on targets rather than concrete restrictions on length of tenancy. Change “maximum” into “average” or “expected”. | 11 | What is the average length of stay/average length of time your service will expect to work with a service user? |
| Amendment | 12 | If so, what is it? | See above. | 12 | What is the expected maximum length of stay/time? |
| New Item | - | - | Suggested by PMG | 22 | Can residents/service users control the temperature in their rooms (e.g. heating/cooling)? |
| Amendment | 28 | Do non-detained patients/residents have a key or entry code to the front door of the facility? | Remove distinction between detained’ and non-detained residents; item should reflect access arrangements for all residents. Service users with short-hold tenancies (supported housing) must, legally, have full access to their property. | 29 | Do residents/service users have a key or entry code to the front door of the project? |
| Amendment | 39 | Which organisation(s) employ(s) your staff?   - Public service - health [N/Y] - Public service – social services [N/Y] - Independent/private organisation[N/Y] - Voluntary organisation (e.g. NGO)[N/Y] | Add response option: “Housing association”. Add “(e.g. charities)” to response option “Voluntary organisation (e.g. NGO)” | 40 | Which organisation(s) employ(s) your staff?   - Public service - health [N/Y] - Public service – social services [N/Y] - Independent/private organisation[N/Y] - Housing association [N/Y] - Voluntary organisation/charity [N/Y] |
| Amendment | 43 | We know it is not always possible to keep staff up to date with new developments but we are interested in knowing what types of training the staff in your unit have received. In which of the following areas have your staff received FORMAL training in the last 12 months and how many staff members received this training?  Number of staff that received training for:   - Patients’ rights - Mental health law - Communication skills - Recovery based practice - Reporting of adverse incidents - De-escalation techniques - Restraint and control technique - Talking therapies (e.g. counselling, psychotherapy - Cognitive Behavioural Therapy - Family work (e.g. psychoeducation) - Health promotion (e.g. exercise and diet) - Smoking cessation - Alcohol and drug misuse - Work skills and employment - Other - please describe | Remove “restraint and control techniques”. Add response options: “Self-harm and suicide”, “Breakaway techniques”, “Food handling” , “Health and safety”, ‘Safeguarding”, “Vulnerable adults”, “Medication training”, “Motivational interviewing”, “Client empowerment and involvement”, “Support planning”, “Risk assessment”,  “Communication skills”, “Motivational communication” and “relationship building” | 44 | We know it is not always possible to keep staff up to date with new developments but we are interested in knowing what types of training the staff in your unit have received. In which of the following areas have your staff received FORMAL training in the last 12 months and how many staff members received this training?  Number of staff that received training for:   - Risk assessment (incl. suicide/self-harm) - Communication skills - Welfare and benefits - Mental health awareness - Recovery based practice - Reporting of adverse incidents - De-escalation and breakaway techniques - Family work (e.g. psychoeducation) - Health promotion (e.g. exercise and diet) - Smoking cessation - Alcohol and drug misuse - Work skills and employment - Medication management - Patients’ rights - Support planning - Mental health law - Food handling/basic food hygiene - Other – please describe |
| New Item |  | - | Multiple general comments from focus groups and expert panels regarding the training and expertise of staff. This item proposed, discussed and agreed by PMG. | 45 | How many of your staff are qualified to at least Level 2 NVQ in Health and Social Care (Adults)? |
| Amendment | 46 | Approximately how many of your patients/residents will move on to more independent accommodation in the next 2 years? | Replace “independent accommodation” with “independence” or “less support” (floating outreach clients should already be in independent accommodation). | 48 | *For accomodation-based services:*  Approximately how many of your service users will move on to more independent accommodation in the next 2 years?  *For floating support services:*  Approximately how many of your service users will successfully move on from your service in the next 2 years? |
| Amendment | 47 | Please estimate the number of your patients/residents who have moved on from your unit to more independent accommodation in the last 2 years? | See above. | 49 | *For accomodation-based services:*  Please estimate the number of your service users who have moved on from your service to more independent accommodation in the last 2 years?  *For floating support services:*  Please estimate the number of your service users who have successfully moved on from your service in the last 2 years? |
| Amendment | 50 | Do all patients/residents have an allocated worker (key worker/primary nurse/named nurse/personal counsellor) in your unit? | “Keyworker” is the most appropriate term; remove other options. | 52 | Do all residents/service users have an allocated worker (key worker/support worker) in your project/service? |
| Amendment | 51 | If yes, how often do they usually meet one-to-one? | Change item to reflect ‘formal keywork sessions’; informal, non-therapeutic, one-on-one contact is common in residential settings. | 53 | If yes, how often do they usually meet for a formal / planned one-to-one session? |
| New Item | - | - | Focus group comments for Item 56 (“How often do you have meetings where staff and residents/service users discuss the running of the project/service?”) emphasised the need to better involve service users in the running of the project and getting their feedback; this item was created in response to this. This item proposed, discussed and agreed by PMG. | 59 | Is it chaired or co-chaired by a resident/service user?  [*Note: This relates to Item 56 – “How often do you have meetings where staff and residents/service users discuss the running of the project/service?”*] |
| Amendment | 57 | To what extent do residents’ views influence decision-making in your facility?  [Hardly at all, A little, Moderately, Quite a lot, A great deal] | Item is likely to encourage false responses from managers due to the global nature of the question.  Add response options that reflect residents influence in particular areas, such as ‘Décor’, ‘Selection of keyworker’, ‘Staff recruitment’, ‘Changes to risk assessments’,  ‘Meal options’, and ‘Entertainment’ | 60-64 | To what extent do residents’/service users’ views influence decision-making in the following areas:   - Décor (Item 60) - Entertainment (Item 61) - Selection of keyworker (Item 62) - Changes to risk assessments (Item 63) - Staff recruitment (Item 64)   [Hardly at all, A little, Moderately, Quite a lot, A great deal] |
| New Item | - | - | Focus group comments on Q57: Add ‘What is the forum for service users to have their views heard?’ or ‘What are the methods of eliciting service users views and do they result in influencing decisions?’ | 65 | Does your organisation carry out a survey of your residents’/service users’ views of your service at least annually |
| New Item | - | - | See above | 66 | If yes, are the results fed back to the residents/service users, please describe? |
| New Item | - | - | See above | 67 | If yes, please describe how are the results fed back? |
| Amendment | 58 | Who reviews therapeutic effects and side effects of psychiatric medication?   - Service users - Staff | Medication is reviewed by GPs or psychiatrists. Project staff typically monitor rather than review effects and side effects. | 68 | Who reviews therapeutic effects and side effects of psychiatric medication most in your service?   - Residents/service users - Staff - Outside staff (e.g. GP, psychiatrist, care co-ordinator) |
| Amendment | 60 | Do patients/residents here who are prescribed antipsychotic medication have the following investigations to check for side effects at least annually?   - Weight checks, - Blood tests (e.g. lipids, glucose), - ECGs   [Yes, No] | Managers may find it difficult to answer this item; GPs do not often inform staff when reviews occur.  Add additional response option: “Not known”. | 70 | Do residents/service users here who are prescribed antipsychotic medication have the following investigations to check for side effects at least annually?   - Weight checks, - Blood tests (e.g. lipids, glucose), - ECGs   [Yes, No, Not known] |
| Amendment | 62 | What does your facility do to promote physical health among patients/residents with regard to the following areas:  Smoking?   - Smoking cessation - Other (please describe):   Diet?   - Dietary advice - Support with cooking/choosing healthier meals - Other (please describe):   Physical exercise?   - Exercise advice - Support to access physical exercise e.g. gym/swimming pool etc - Other (please describe):   Sexual health?   - Supporting access to or giving sexual health information - Supporting access to or giving advice about contraception - Providing condoms - Other (please describe):   Dental care?   - Arranging or encouraging dental check-ups - Other (please describe): |  | 70 | What does your project/service do to promote physical health among residents/service users with regard to the following areas:  Smoking?   - Formal smoking cessation - Other (e.g. rationing cigarettes) (please describe):   Diet?   - Dietary advice - Support with cooking/choosing healthier meals - Other (e.g. referral to a dietician, nutritionist, GP, etc.) (please describe):   Physical exercise?   - Exercise advice - Support to access and attend physical exercise (e.g. gym, swimming pool, walking group etc.) - Other (e.g. referral to GP or signposting to local gyms and sporting venues, etc.) (please describe):   Sexual health?   - Supporting access to or giving sexual health information - Supporting access to or giving advice about contraception - Providing condoms - Other (please describe):   Dental care?   - Arranging or encouraging dental check-ups - Other (please describe): |
| Amendment | 65 | Imagine you have a patient who is becoming agitated, what would you do? (Researcher to tick off appropriate responses e.g. de-escalation techniques then to say… And what if that did not work?.... What do you do after the incident?)  And if that did not work? (Researcher to tick off appropriate responses e.g. offering oral medication, using restraint, using seclusion and in community settings, calling for support from the police. Then researcher to keep asking)  And if that didn’t work? (Researcher to tick off appropriate responses. Then researcher to keep asking)  What do you do after the incident? (Researcher to tick off appropriate responses e.g. making a note in the file and holding a debriefing meeting with staff and with patients/residents)  (Open ended question. DO NOT SHOW MANAGER THE LIST)   - Noticing early signs of agitation - Use of de-escalation techniques - Offering oral medication - Calling a doctor or senior member of staff - Use of a seclusion room/suite - Physical restraint by team - Physical restraint using equipment (e.g. mechanical restraints) - If so, what equipment is used? - Use of injectable medication - Calling the police or security staff - Recording events in case notes - Review of continuing need for restraint or seclusion - Holding a debriefing meeting for staff - Holding a debriefing meeting for patients/residents | Remove: “Physical restraint using equipment”, “Use of a seclusion room/suite”, “Use of injectable medication” and “Review of continuing need for restraint or seclusion”, “Offering oral medication”, “Physical restraint by team”.  Change: “Noticing early signs of agitation” to “Noticing early signs of distress” or “Noticing  relapse indicators”. “Calling a doctor or senior member of staff” to “Calling care co-ordinator”, “Calling crisis team” or “Calling home treatment team”. “Calling the police or security staff” to “Calling the police”.  Add: “Incident reports” and “Basic counselling”. | 75 | Imagine you have a resident/service user who is becoming agitated, what would you do? (Researcher to tick off appropriate responses e.g. de-escalation techniques then to say)  And if that did not work? (Researcher to tick off appropriate responses e.g. offering oral medication, calling for support from the police. Then researcher to keep asking)  And if that didn’t work? (Researcher to tick off appropriate responses. Then researcher to keep asking)  What do you do after the incident? (Researcher to tick off appropriate responses e.g. making a note in the file and holding a debriefing meeting with staff and with residents/service users)  (Open ended question. DO NOT SHOW MANAGER THE LIST)   - Noticing early signs of agitation - Use of de-escalation techniques - Ensuring other staff, service users or others are safe - Offering oral medication - Calling a senior member of staff - Calling the police / security service - Recording events in case notes - Update risk assessment - Holding a debriefing meeting for staff - Holding a debriefing meeting for residents/service users - Informing other relevant professionals - Completing incident reports |
| Amendment | 67 | How many of your staff are trained in control and restraint techniques? | Change “control and restraint techniques” to “breakaway techniques”. | 77 | How many of your staff are trained in breakaway techniques? |
| Amendment | 68 | How many times has physical restraint or seclusion been used in the unit in the last 3 months? | Change item to: ‘How many times have the police been called in the last 3 months?’, ‘How many incident reports relating to aggressive behaviour have been submitted in the last 3 months?’ or “How many times have the police been called to deal with agitated patient in the last 3 months?”. Or specify, “How many times have you had to deal with incidents: -fire -self harm, -physical aggression, etc… in the last 3 months?” | 78 | How many times has an incident report related to aggressive behaviour been completed in the last 3 months? |
| New Item |  | - | See above. | 79 | How many times have the police been called to deal with an aggressive resident/service user in the last 3 months? |
| Deletion | 69 | How many patients/residents are currently receiving:  Typical antipsychotic medications? | Medication variables are now assessed in new items 80-84. | - | - |
| Deletion | 70 | How many patients/residents are currently receiving:  Atypical antipsychotic medications? | As above. | - | - |
| Deletion | 71 | How many patients/residents are currently receiving:  Clozapine? | As above. | - | - |
| Deletion | 72 | How many patients/residents are currently receiving:  More than two antipsychotics? | As above. | - | - |
| Deletion | 73-75 | How far do residents’/patients’ views about the following influence the prescribing of medication?   - Dose of medication - Changing medication - Stopping medication | Item refers to prescribing practices; managers may be unable to answer these items. Also, the utility of item is questionable; prescribers are independent of supported accommodation and vary in their willingness to include patient views in their prescribing practices. Consider replacing with “How do you support your customers with their medication?” or “How do you support your customers to request a review of medication?”.  Medication variables are now assessed in new items 80-84. | - | - |
| New Item | - | - | PMG agreed that items about specific interventions were not appropriate (Items 69-75), but facilitation of access to treatment and interventions should be captured. Items relating to the expected level of support with medication were developed by the research team, externally validated by members of Expert Panel then agreed by the PMG. | 80-84 | How many of your residents/service users require staff support in the following areas:   - Administration of medication? (Item 80) - Prompting to take medication? (Item 81) - Prompting to attend for depot antipsychotic appointments? (Item 82) - Prompting to attend for clozapine related blood tests? (Item 83) - Regular checks on medication adherence (e.g. dosette review) (Item 84) |
| Deletion | 81 | How many of your staff have had adequate training in family psychoeducation to provide it to your patients/residents and their families? | Inappropriate for setting; staff not generally trained in providing “psychoeducation”. | - | - |
| Deletion | 82 | How many families of your current patients/residents have had family psychoeducation in the last 12 months? | As above | - | - |
| Deletion | 83 | How many psychoeducation meetings are usually offered (either by staff from the unit or elsewhere)? | As above | - | - |
| Deletion | 84 | Are there any other family interventions available to your residents in the unit or elsewhere? | As above | - | - |
| Deletion | 85 | How many of your staff have had adequate training in CBT to provide it to your patients/residents? | Inappropriate for setting; staff not generally trained in providing “CBT”. | - | - |
| Deletion | 86 | How many of your current patients/residents have had CBT in the last 12 months? (In the unit? Elsewhere?) | As above | - | - |
| Deletion | 87 | How many CBT appointments are usually offered either by staff from the unit or elsewhere? | As above | - | - |
| Deletion | 91 | How many of your current patients/residents attend any of the following:  A sheltered workshop? | Remove “sheltered workshop”. | - | - |
| Deletion | 94 | Other employment/training schemes or courses (please describe) | Remove as duplicative of other items on supported employment/courses | - | - |
| Deletion | 99 | How many of your patients/residents have accessed any other organisation (not listed in q. 89-94) in the last 12 months to help them get mainstream work (such as an employment agency)? | Remove as duplicative of other items on supported employment/courses | - | - |
| Amendment | 102 | What things do your staff do to support patients’/residents’ programmed activities?   - Make links with local entertainment venues such as cinemas, theatres, concert halls - Make links with local cafes and restaurants - Make links with local sports facilities - Make links with places of worship and religious organisations - Make links with local neighbourhood organisations (e.g. local tenants’ groups), - Provide regular activities in the **unit** for patients/residents - Provide the same programme of activities for all patients/residents - Provide different programme of activities for each patients - Other - please describe   *[N/Y]* | Term “programmed activities” is not appropriate.  Consider adding response items: “Day centres”, ”Beauty services”, ”Education/courses”, “Art groups”, “Walking groups”, “Coffee mornings”, “Film club”, “Cookery groups”. | 101 | What things do your staff do to support residents’/service users’ activities?   - Make links with local entertainment venues such as cinemas, theatres, concert halls - Make links with local cafes and restaurants - Make links with local sports facilities - Make links with places of worship and religious organisations - Make links with local neighbourhood organisations (e.g. local tenants’ groups) - Provide regular activities in the project/service for residents’/service users - Provide the same programme of activities for all residents’/service users - Provide different programme of activities for each resident/service user - Other - please describe   *[N/Y]* |
| Deletion | 105 | Are any other treatments or interventions available to your patients/residents that we have not mentioned? | Remove as duplicative of other items |  | - |
| Deletion | 106 | If so, what? (Please note any other interventions mentioned by the interviewee) | Remove as not applicable as item 105 deleted |  | - |
| Amendment | 108 | If yes, in addition to staff, who is involved in creating the plan? (tick all that apply):   - Patient/resident - Family member/carer - Community agencies | Note: Terminology changed to be consistent with previous items | 105 | If yes, in addition to staff, who is involved in creating the plan? (tick all that apply):   - Resident/service user - Family member/carer - NHS community team care co-ordinator |
| Amendment | 109-111 | In general, when reviewing your patients/residents’ progress and care e.g. at a ward round or care review meeting:   - Are discussions held with the patient/resident beforehand to identify their priorities? - Is the patient/resident usually present at the meeting? - Is the meeting usually multidisciplinary? | Replace “ward round” with “care plan review” or “support plan review”. | 106-108 | In general, when reviewing your residents’/service users’ progress and care (e.g. at a care review / CPA meeting):   - Are discussions held with the patient/resident beforehand to identify their priorities? - Is the patient/resident usually present at the meeting? - Is the meeting usually multidisciplinary? |
| New Item | - | - | Add item relating to personal budgets. | 112-113 | Do your residents/service users have personal budgets (health or social care funded)? (Item 112)  If yes, please describe (Item 113) |
| Amendment | 115 | Are non-detained patients/residents free to have consensual sexual relationships in the unit? | Remove distinction between detained and non-detained patients. | 114 | Are residents/service users free to have consensual sexual relationships in the project/service? |
| Deletion | 116 | Are patients/residents free to send and receive uncensored mail or email? | Item removed as irrelevant – no services reported any censoring of mail/email |  | - |
| Amendment | 122 | Are your detained patients/residents routinely given written information about their civil rights? | Delete “detained”, services listed in 122-125 are provided for all service users, including service users with CTOs. | 120 | Are your residents/service users under CTO/Section 41 routinely given written information about their civil rights? |
| Deletion | 125 | Are your non-detained patients/residents routinely given written information about their civil rights? | The notion of “routine” provision of civil rights information not relevant |  | - |
| Amendment | 127 | What PERCENTAGE of your patients/residents who have legal capacity will be assisted to vote in the next political election? | The majority of service users will have capacity; consider changing item to “Are clients encouraged to vote?” or “Are clients encouraged to register to vote?” | 124 | Roughly what PERCENTAGE of your residents/service users will be assisted to vote in the next political election? |
| Amendment | 128 | Do all your clinical staff have a named supervisor? | Replace “clinical staff” with “staff” or “support staff” or “staff (non-clinical)” | 125 | Do all your clinical and support staff have a named supervisor? |
| Amendment | 132 | Does this unit have an external organisational review of care? | Add additional component to item: “If so, who?” | 129 | Does this project/service have an external organisational review/inspection of care? |
| New Item | - | - | See 132 above | 131 | If yes, which organisation carried out the inspection? |
| Deletion | 140 | What sort of unit is it? (tick one answer that applies)  *[A ward in a hospital, A community based facility, A house/unit within hospital grounds (not a ward), Other (please describe)]* | Deleted as Item 9 captures the type of supported accommodation service provided | - | - |
