# Supplementary material for: Adaptation of the Quality Indicator for Rehabilitative Care (QuIRC) for use in mental health supported accommodation services (QuIRC-SA)
Source: BMC Psychiatry. 2016 Apr 14;16:101. doi: 10.1186/s12888-016-0799-4 (PMC4831104; doi:10.1186/s12888-016-0799-4)
Supplement: Additional file 2: — Table S1 contains results of the item level inter-rater reliability analyses of the adapted QuIRC. (DOCX 62 kb) [file 12888_2016_799_MOESM2_ESM.docx]

**On-line Supplementary Table. Inter-rater reliability of the adapted QuIRC items**

*Legend*

|  | Descriptive item. | |  |  |  |  |  |  |  |  |
| --- | --- | --- | --- | --- | --- | --- | --- | --- | --- | --- |
|  | Unable to complete analysis. | |  |  |  |  |  |  |  |  |
|  | Poor reliability (ICC/Kappa <0.75/0.8) | |  |  |  |  |  |  |  |  |
|  | **Item description** | **Scored Item?** | | **Statistic** | **Value** | **95% CI Lower** | **95% CI Upper** | ***N*** | **Range** | **Mean** |
| **Item 1** | *Total beds / places* | N | | ICC | 1.00 | - | - | 51 | 3-150 | 24.45 |
| **Item 2** | *Beds / places currently filled* | N | | ICC | 1.00 | - | - | 52 | 3-150 | 24.17 |
| **Item 3** | *Number of males* | N | | ICC | 1.00 | - | - | 51 | 0-60 | 12.94 |
| **Item 4** | *Number of females* | N | | ICC | 1.00 | - | - | 51 | 0-90 | 10.63 |
| **Item 5** | *Number of SUs detained involuntarily* | N | | ICC | 1.00 | - | - | 49 | 0-12 | 1.76 |
| **Item 6** | *Number of support/clinical staff working in unit* | Y | | ICC | 1.00 | - | - | 52 | 1-42 | 9.63 |
| **Item 7 – A** | *Psychiatrist* | Y | | Kappa | 1.00 | - | - | 48 | - | - |
| **Item 7 - AA** | *Psychiatrist FTE* | Y | | Too few cases for analysis (N=1) | | | | 1 | 0.2-0.2 | 0.20 |
| **Item 7 – B** | *Clinical psychologist* | Y | | Kappa | 1.00 | - | - | 47 | - | - |
| **Item 7 - BB** | *Clinical psychologist FTE* | Y | | ICC | 1.00 | - | - | 2 | 0.5-0.6 | 0.55 |
| **Item 7 – C** | *Occupational therapist* | Y | | Kappa | 1.00 | - | - | 46 | - | - |
| **Item 7 - CC** | *Occupational therapist FTE* | Y | | ICC | 1.00 | - | - | 3 | 0.4-0.8 | 0.57 |
| **Item 7 – D** | *Nurse* | Y | | Kappa | 1.00 | - | - | 47 | - | - |
| **Item 7 - DD** | *Nurse FTE* | Y | | ICC | 1.00 | - | - | 3 | 1-15 | 7.67 |
| **Item 7 – E** | *Support worker* | Y | | No analysis. Variables are constants | | | | 47 |  |  |
| **Item 7 - EE** | *Support worker FTE* | Y | | ICC | 1.00 | 1.00 | 1.00 | 47 | 1-39 | 7.29 |
| **Item 7 – F** | *Social worker* | Y | | Kappa | 1.00 | - | - | 48 | - | - |
| **Item 7 - FF** | *Social worker FTE* | Y | | Too few cases for analysis (N=1) | | | | 1 | 1-1 | 1.00 |
| **Item 7 – G** | *Counsellor/psychotherapist* | Y | | Kappa | 1.000 | - | - | 48 | - | - |
| **Item 7 - GG** | *Counsellor/psychotherapist FTE* | Y | | Too few cases for analysis (N=0) | | | | 0 | - | - |
| **Item 7 – H** | *Volunteer* | Y | | Kappa | 1.00 | - | - | 46 | - | - |
| **Item 7 - HH** | *Volunteer FTE* | Y | | ICC | 1.00 | - | - | 7 | 0.1-39 | 6.17 |
| **Item 7 – I** | *Art therapist* | Y | | Kappa | 1.00 | - | - | 47 | - | - |
| **Item 7 – II** | *Art therapist FTE* | Y | | Too few cases for analysis (N=1) | | | | 1 | 0.2-0.2 | 0.20 |
| **Item 7 – J** | *Drug and alcohol worker* | New | | Kappa | 1.00 | - | - | 47 | - | - |
| **Item 7 - JJ** | *Drug and alcohol worker FTE* | New | | Scale has zero variance items | | | | 2 | 1-1 | 1.00 |
| **Item 7 – K** | *Other* | Y | | Kappa | 0.83 | 0.64 | 1.00 | 45 | - | - |
| **Item 7 - KK** | *Other FTE* | Y | | Too few cases for analysis (N=0) | | | | 0 | - | - |
| **Item 8** | *Number of staff in substantive posts* | N | | ICC | 1.00 | - | - | 52 | 1-42 | 8.60 |
| **Item 9 – A** | *Level of suppport* | Y | | Kappa | 0.97 | 0.93 | 1.00 | 52 | - | - |
| **Item 9 – B** | *FS - Average contact hours per week* | N | | ICC | 1.00 | - | - | 17 | 1-19 | 6.18 |
| **Item 10** | *Years unit open in current form* | N | | ICC | 1.00 | - | - | 51 | 0.2-30 | 11.06 |
| **Item 11** | *Average length of stay (years)* | New | | ICC | 1.00 | - | - | 38 | 0.5-7 | 2.33 |
| **Item 12** | *Expected max. length of stay (years)* | N | | ICC | 1.00 | - | - | 31 | 0.5-5 | 2.05 |
| **Item 13** | *Condition of building outside* | Y | | Kappa | 1.00 | - | - | 52 | - | - |
| **Item 14** | *Condition of decor indoors* | Y | | Kappa | 1.00 | - | - | 52 | - | - |
| **Item 15** | *General cleanliness indoors* | Y | | Kappa | 1.00 | - | - | 52 | - | - |
| **Item 16 - A** | *No access outside space* | Y | | Kappa | 1.00 | - | - | 52 | - | - |
| **Item 16 - B** | *Access to garden* | Y | | Kappa | 0.96 | 0.87 | 1.00 | 52 | - | - |
| **Item 16 - C** | *Access to patio* | Y | | Kappa | 1.00 | - | - | 51 | - | - |
| **Item 16 - D** | *Access to balcony* | Y | | Kappa | 1.00 | - | - | 52 | - | - |
| **Item 16 - E** | *Access to refuse/delivery area* | Y | | Kappa | 1.00 | - | - | 51 | - | - |
| **Item 17** | *Adequacy of outside space* | Y | | Kappa | 1.00 | - | - | 52 | - | - |
| **Item 18 - A** | *Newspaper provided* | Y | | Kappa | 1.00 | - | - | 52 | - | - |
| **Item 18 - B** | *Computers with internet provided* | Y | | Kappa | 1.00 | - | - | 52 | - | - |
| **Item 18 - C** | *Access to telephone provided* | Y | | Kappa | 1.00 | - | - | 51 | - | - |
| **Item 19** | *Number of single bedrooms* | Y | | ICC | 1.00 | - | - | 36 | 3-36 | 12.06 |
| **Item 20** | *Max number in shared room* | Y | | ICC | 1.00 | 1.00 | 1.00 | 36 | 1-2 | 1.08 |
| **Item 21 - A** | *Choose colour of walls* | Y | | Kappa | 1.00 | - | - | 51 | - | - |
| **Item 21 - B** | *Choose soft furnishings* | Y | | Kappa | 1.00 | - | - | 52 | - | - |
| **Item 21 - C** | *Choose wall decorations* | Y | | Kappa | 1.00 | - | - | 52 | - | - |
| **Item 21 - D** | *Choose furniture* | N | | Kappa | 1.00 | - | - | 52 | - | - |
| **Item 22** | *Control temperature* | N | | Kappa | 1.00 | - | - | 52 | - | - |
| **Item 23** | *Lockable storage* | Y | | Kappa | 1.00 | - | - | 52 | - | - |
| **Item 24 - A** | *Single sex areas?* | N | | Kappa | 1.00 | - | - | 49 | - | - |
| **Item 24 - B** | *Single sex bedrooms* | N | | Kappa | 1.00 | - | - | 48 | - | - |
| **Item 24 - C** | *Single sex bathroom* | N | | Kappa | 1.00 | - | - | 48 | - | - |
| **Item 24 - D** | *Single sex toilet* | N | | Kappa | 1.00 | - | - | 48 | - | - |
| **Item 25** | *Lock bathroom door* | Y | | Kappa | 1.00 | - | - | 52 | - | - |
| **Item 26** | *Lock toilet door* | Y | | Kappa | 1.00 | - | - | 52 | - | - |
| **Item 27** | *Set time for visitors* | Y | | Kappa | 1.00 | - | - | 52 | - | - |
| **Item 28 - A** | *Room for visits* | Y | | Kappa | 0.97 | 0.91 | 1.00 | 52 | - | - |
| **Item 28 - B** | *Specific visitors' room* | Y | | Kappa | 1.00 | - | - | 52 | - | - |
| **Item 29** | *Key/code to front door* | Y | | Kappa | 1.00 | - | - | 52 | - | - |
| **Item 30** | *Key to bedroom* | Y | | Kappa | 1.00 | - | - | 52 | - | - |
| **Item 31** | *Meals cooked central kitchen* | Y | | Kappa | 1.00 | - | - | 52 | - | - |
| **Item 32** | *Quality of cooked meals* | N | | Kappa | 1.00 | - | - | 28 | - | - |
| **Item 33** | *Choice of meals* | Y | | Kappa | 1.00 | - | - | 29 | - | - |
| **Item 34** | *Facilities for making meal/snack* | Y | | Kappa | 1.00 | - | - | 52 | - | - |
| **Item 35** | *Facility accessible 24hrs* | Y | | Kappa | 1.00 | - | - | 52 | - | - |
| **Item 36** | *SUs usually prepare own meals* | Y | | Kappa | 1.00 | - | - | 52 | - | - |
| **Item 37** | *Facilities to do own laundry* | Y | | Kappa | 1.00 | - | - | 52 | - | - |
| **Item 38** | *SUs with physical disabilities* | N | | ICC | 1.00 | - | - | 35 | 0-4 | 0.66 |
| **Item 39** | *Facilities for SUs with phys. disabilities* | Y | | Kappa | 0.98 | 0.93 | 1.00 | 52 | - | - |
| **Item 40 - A** | *Public service - Health* | N | | Kappa | 1.00 | - | - | 52 | - | - |
| **Item 40 - B** | *Public service - Social services* | N | | Kappa | 1.00 | - | - | 52 | - | - |
| **Item 40 - C** | *Independent/private organisation* | N | | Kappa | 0.945 | 0.84 | 1.00 | 52 | - | - |
| **Item 40 - D** | *Housing association* | New | | Kappa | 1.00 | - | - | 52 | - | - |
| **Item 40 - E** | *Voluntary organisation/charity* | N | | Kappa | 0.96 | 0.88 | 1.00 | 52 | - | - |
| **Item 41** | *Ex-service users employed* | N | | Kappa | 1.00 | - | - | 52 | - | - |
| **Item 42** | *Ex-service user job (description)* | N | | Descriptive item. No analysis. | | | |  |  |  |
| **Item 43** | *Ex-sevice user on payroll* | N | | Kappa | 1.00 | - | - | 15 | - | - |
| **Item 44 - A** | *Safeguarding* | New | | Kappa | 1.00 | - | - | 52 | - | - |
| **Item 44 - AA** | *Staff trained in safeguarding* | New | | ICC | 1.00 | - | - | 48 | 1-34 | 7.27 |
| **Item 44 - B** | *Risk assessment* | New | | Kappa | 1.00 | - | - | 52 | - | - |
| **Item 44 - BB** | *Staff trained in risk assessment* | New | | ICC | 1.00 | - | - | 32 | 1-34 | 6.66 |
| **Item 44 - C** | *Communication skills training* | Y | | Kappa | 1.00 | - | - | 49 | - | - |
| **Item 44 - CC** | *Staff trained in communication skills* | Y | | ICC | 1.00 | - | - | 20 | 1-34 | 7.20 |
| **Item 44 - D** | *Welfare and benefits* | Y | | Kappa | 1.00 | - | - | 51 | - | - |
| **Item 44 - DD** | *Staff trained in welfare and benefits* | Y | | ICC | 1.00 | - | - | 28 | 1-23 | 4.82 |
| **Item 44 - E** | *Mental health awareness* | Y | | Kappa | 1.00 | - | - | 51 | - | - |
| **Item 44 - EE** | *Staff trained in MH awareness* | Y | | ICC | 1.00 | - | - | 38 | 1-34 | 7.18 |
| **Item 44 - F** | *Recovery based practice* | Y | | Kappa | 1.00 | - | - | 52 | - | - |
| **Item 44 - FF** | *Staff trained in recov. based practice* | Y | | ICC | 1.00 | - | - | 30 | 1-34 | 7.23 |
| **Item 44 - G** | *Reporting of adverse events* | Y | | Kappa | 1.00 | - | - | 51 | - | - |
| **Item 44 – GG** | *Staff trained in reporting of AE* | Y | | ICC | 1.00 | - | - | 33 | 1-34 | 6.33 |
| **Item 44 - H** | *De-escalation / breakaway techniques* | Y | | Kappa | 1.00 | - | - | 52 | - | - |
| **Item 44 - HH** | *Staff trained in de-escalation / breakaway* | Y | | ICC | 1.00 | - | - | 21 | 1-23 | 6.90 |
| **Item 44 – I** | *Family work training* | Y | | Kappa | 1.00 | - | - | 52 | - | - |
| **Item 44 - II** | *Staff trained in family work* | Y | | ICC | 1.00 | - | - | 6 | 1-12 | 4.50 |
| **Item 44 - J** | *Health promotion training* | Y | | Kappa | 1.00 | - | - | 52 | - | - |
| **Item 44 - JJ** | *Staff trained in health promotion* | Y | | ICC | 1.00 | - | - | 21 | 1-23 | 6.57 |
| **Item 44 - K** | *Smoking cessation training* | Y | | Kappa | 1.00 | - | - | 52 | - | - |
| **Item 44 - KK** | *Staff trained in smoking cessation* | Y | | ICC | 1.00 | - | - | 17 | 1-12 | 4.71 |
| **Item 44 - L** | *Alcohol and drug misuse training* | Y | | Kappa | 1.00 | - | - | 52 | - | - |
| **Item 44 - LL** | *Staff trained in alcohol and drug misuse* | Y | | ICC | 1.00 | - | - | 23 | 1-23 | 6.57 |
| **Item 44 - M** | *Work skills and employment training* | Y | | Kappa | 1.00 | - | - | 52 | - | - |
| **Item 44 – MM** | *Staff trained in work skills and employment* | Y | | ICC | 1.00 | - | - | 12 | -1-12 | 3.67 |
| **Item 44 - N** | *Medication management* | New | | Kappa | 1.00 | - | - | 52 | - | - |
| **Item 44 - NN** | *Staff trained in medication management* | New | | ICC | 1.00 | - | - | 27 | 1-34 | 8.67 |
| **Item 44 - O** | *Patient rights* | Y | | Kappa | 1.00 | - | - | 52 | - | - |
| **Item 44 – OO** | *Staff trained in patient rights* | Y | | ICC | 0.97 | 0.94 | 0.99 | 23 | 1-23 | 5.96 |
| **Item 44 - P** | *Support planning* | New | | Kappa | 1.00 | - | - | 52 | - | - |
| **Item 44 - PP** | *Staff trained in support planning* | New | | ICC | 1.00 | - | - | 37 | 1-34 | 7.16 |
| **Item 44 - Q** | *Mental health law* | Y | | Kappa | 1.00 | - | - | 52 | - | - |
| **Item 44 – QQ** | *Staff trained in mental health law* | Y | | ICC | 1.00 | - | - | 31 | 0-34 | 6.29 |
| **Item 44 - R** | *Food handling / basic food hygiene* | New | | Kappa | 1.00 | - | - | 50 | - | - |
| **Item 44 - RR** | *Staff trained in food handling* | New | | ICC | 1.00 | 0.99 | 1.00 | 30 | 0-34 | 5.87 |
| **Item 44 - S** | *Other* | Y | | Kappa | 0.96 | 0.88 | 1.00 | 52 | - | - |
| **Item 44 - SS** | *Staff trained in other* | Y | | ICC | 0.98 | 0.75 | 1.00 | 3 | 2-12 | 5.33 |
| **Item 44 - T** | *Description of other* | N | | Descriptive item. No analysis. | | | |  |  |  |
| **Item 45** | *Number of staff qualified to NVQ Level 2* | New | | ICC | 1.00 | - | - | 47 | 0-25 | 7.06 |
| **Item 46** | *Staff left in last 2 years* | N | | ICC | 1.00 | - | - | 49 | 0-12 | 2.16 |
| **Item 47** | *Hopeful SUs will move on* | Y | | Kappa | 1.00 | - | - | 52 | - | - |
| **Item 48** | *Number of SUs that will move on* | Y | | Kappa | 1.00 | - | - | 52 | - | - |
| **Item 49** | *Number SUs have moved on (last 2 years)* | Y | | ICC | 1.00 | - | - | 48 | 0-400 | 34.15 |
| **Item 50 - A** | *Able to do most things w/o assistance* | N | | ICC | 1.00 | - | - | 47 | 0-55 | 7.60 |
| **Item 50 - B** | *Able to do some things w/o assistance* | N | | ICC | 1.00 | - | - | 47 | 0-35 | 7.91 |
| **Item 50 - C** | *Able to do very little w/o assistance* | N | | ICC | 1.00 | - | - | 48 | 0-104 | 4.21 |
| **Item 51** | *Aim of unit* | Y | | Kappa | 1.00 | - | - | 52 | - | - |
| **Item 52** | *All patients have allocated keyworker* | Y | | Kappa | 1.00 | - | - | 52 | - | - |
| **Item 53** | *Frequency of 1:1 meetings with keyworker* | Y | | Kappa | 1.00 | - | - | 51 | - | - |
| **Item 54** | *Patients have access to staff office* | N | | Kappa | 1.00 | - | - | 50 | - | - |
| **Item 55** | *Staff only toilets* | N | | Kappa | 1.00 | - | - | 52 | - | - |
| **Item 56** | *Staff only kitchen* | N | | Kappa | 1.00 | - | - | 51 | - | - |
| **Item 57** | *Staff only break room* | N | | Kappa | 1.00 | - | - | 51 | - | - |
| **Item 58** | *Frequency of facility meetings* | Y | | Kappa | 0.97 | 0.92 | 1.00 | 52 | - | - |
| **Item 59** | *Meeting chaired by SU* | N | | Kappa | 1.00 | - | - | 45 | - | - |
| **Item 60** | *SU influence decision making - Decor* | New | | Kappa | 1.00 | - | - | 50 | - | - |
| **Item 61** | *SU influence decision making - Entertainment* | New | | Kappa | 1.00 | - | - | 51 | - | - |
| **Item 62** | *SU influence decision making - Keyworker* | New | | Kappa | 1.00 | - | - | 19 | - | - |
| **Item 63** | *SU influence decision making - Risk Ax* | New | | Kappa | 1.00 | - | - | 50 | - | - |
| **Item 64** | *SU influence decision making - Staff recruitment* | New | | Kappa | 1.00 | - | - | 52 | - | - |
| **Item 65** | *Survey of SU views* | New | | Kappa | 1.00 | - | - | 52 | - | - |
| **Item 66** | *Feedback* | New | | Kappa | 1.00 | - | - | 52 | - | - |
| **Item 67** | *Description - Feedback method* | N | | Descriptive item. No analysis. | | | |  |  |  |
| **Item 68 - A** | *SU medication review* | Y | | Kappa | 1.00 | - | - | 51 | - | - |
| **Item 68 - B** | *Staff medication review* | Y | | Kappa | 1.00 | - | - | 51 | - | - |
| **Item 68 - C** | *Outside staff medication review* | New | | Kappa | 1.00 | - | - | 51 | - | - |
| **Item 69** | *Frequency of medication review* | Y | | Kappa | 1.00 | - | - | 47 | - | - |
| **Item 70 - A** | *Weight checks carried out* | Y | | Kappa | 1.00 | - | - | 51 | - | - |
| **Item 70 - B** | *Blood tests carried out* | Y | | Kappa | 1.00 | - | - | 51 | - | - |
| **Item 70 - C** | *ECGs carried out* | Y | | Kappa | 1.00 | - | - | 51 | - | - |
| **Item 71** | *Who makes doctor appointment* | Y | | Kappa | 0.96 | 0.89 | 1.00 | 52 | - | - |
| **Item 72 - A** | *Smoking cessation* | Y | | Kappa | 0.80 | 0.64 | 0.97 | 52 | - | - |
| **Item 72 - B** | *Other smoking* | Y | | Kappa | 0.74 | 0.52 | 0.95 | 52 | - | - |
| **Item 72 - C** | *Description - Other smoking* | N | | Descriptive item. No analysis. | | | |  |  |  |
| **Item 72 - D** | *Dietary advice* | Y | | Kappa | 0.76 | 0.58 | 0.94 | 52 | - | - |
| **Item 72 - E** | *Cooking/choosing healthier meals* | Y | | Kappa | 0.89 | 0.74 | 1.00 | 52 | - | - |
| **Item 72 - F** | *Other diet* | Y | | **Kappa** | **0.68** | **0.48** | **0.88** | **52** |  |  |
| **Item 72 - G** | *Description - Other diet* | N | | Descriptive item. No analysis. | | | |  |  |  |
| **Item 72 - H** | *Excercise advice* | Y | | Kappa | 0.77 | 0.52 | 1.00 | 52 | - | - |
| **Item 72 – I** | *Access to exercise facilities* | Y | | Kappa | 1.00 | - | - | 52 | - | - |
| **Item 72 - J** | *Other physical exercise* | Y | | **Kappa** | **0.56** | **0.34** | **0.79** | **52** |  |  |
| **Item 72 - K** | *Description - Other physcial exercise* | N | | Descriptive item. No analysis. | | | |  |  |  |
| **Item 72 - L** | *Sexual health information* | Y | | Kappa | 0.88 | 0.77 | 1.00 | 52 | - | - |
| **Item 72 - M** | *Contraception advice* | Y | | Kappa | 0.95 | 0.84 | 1.00 | 52 | - | - |
| **Item 72 - N** | *Providing condoms* | Y | | Kappa | 1.00 | - | - | 52 | - | - |
| **Item 72 - O** | *Other sexual health* | Y | | Kappa | 0.72 | 0.53 | 0.91 | 52 | - | - |
| **Item 72 - P** | *Description - Other sexual health* | N | | Descriptive item. No analysis. | | | |  |  |  |
| **Item 72 - Q** | *Dental check-ups* | Y | | Kappa | 0.87 | 0.69 | 1.00 | 52 | - | - |
| **Item 72 - R** | *Other dental care* | Y | | Kappa | 0.83 | 0.65 | 1.00 | 52 | - | - |
| **Item 72 - S** | *Description - Other dental care* | N | | Descriptive item. No analysis. | | | |  |  |  |
| **Item 73** | *General health check-ups* | N | | Kappa | 0.92 | 0.82 | 1.00 | 52 | - | - |
| **Item 74** | *Protocol for dealing with agitated resident* | Y | | Kappa | 1.00 | - | - | 52 | - | - |
| **Item 75 - A** | *Noticing early signs of agitation* | Y | | Kappa | 0.87 | 0.68 | 1.00 | 52 | - | - |
| **Item 75 - B** | *Use of de-escalation* | Y | | Kappa | 1.00 | - | - | 52 | - | - |
| **Item 75 - C** | *Ensure others are safe* | Y | | Kappa | 0.83 | 0.65 | 1.00 | 52 | - | - |
| **Item 75 - D** | *Offering oral medication* | Y | | Kappa | 0.92 | 0.77 | 1.00 | 52 | - | - |
| **Item 75 - E** | *Calling senior staff member* | Y | | Kappa | 0.96 | 0.89 | 1.00 | 52 | - | - |
| **Item 75 - F** | *Calling the police or security staff* | Y | | Kappa | 1.00 | - | - | 52 | - | - |
| **Item 75 - G** | *Recording event in case notes* | Y | | Kappa | 1.00 | - | - | 52 | - | - |
| **Item 75 - H** | *Update risk assessment* | Y | | Kappa | 0.96 | 0.88 | 1.00 | 52 | - | - |
| **Item 75 – I** | *Staff debriefing* | Y | | Kappa | 1.00 | - | - | 52 | - | - |
| **Item 75 - J** | *SU debriefing* | Y | | Kappa | 0.83 | 0.68 | 0.99 | 52 | - | - |
| **Item 75 - K** | *Informing other professionals* | New | | Kappa | 0.95 | 0.87 | 1.00 | 52 | - | - |
| **Item 75 - L** | *Incident report* | New | | Kappa | 1.00 | - | - | 52 | - | - |
| **Item 76** | *Staff trained in de-escalation* | Y | | ICC | 1.00 | - | - | 51 | 0-34 | 6.14 |
| **Item 77** | *Staff trained in breakaway techniques* | Y | | ICC | 1.00 | 1.00 | 1.00 | 51 | 0-24 | 3.76 |
| **Item 78** | *Number of incident reports - Last 3 months* | Y | | ICC | 1.00 | - | - | 52 | 0-20 | 1.81 |
| **Item 79** | *Police calls (aggression) - Last 3 months* | New | | ICC | 1.00 | - | - | 52 | 0-8 | 0.85 |
| **Item 80** | *Requires support - Administration of medication* | New | | ICC | 1.00 | - | - | 48 | 0-23 | 3.58 |
| **Item 81** | *Requires support - Daily prompting for meds* | New | | ICC | 1.00 | - | - | 48 | 0-99 | 7.27 |
| **Item 82** | *Requires support - Prompting for depot* | New | | ICC | 1.00 | 1.00 | 1.0000 | 47 | 0-23 | 2.19 |
| **Item 83** | *Requires support - Prompting clozapine bloods* | New | | ICC | 1.00 | - | - | 47 | 0-23 | 2.19 |
| **Item 84** | *Requires support - Dosette review* | New | | ICC | 1.00 | - | - | 47 | 0-35 | 5.28 |
| **Item 85 - A** | *Self-care support* | Y | | Kappa | 1.00 | - | - | 52 | - | - |
| **Item 85 - B** | *Cleaning support* | Y | | Kappa | 1.00 | - | - | 52 | - | - |
| **Item 85 - C** | *Cooking support* | Y | | Kappa | 1.00 | - | - | 52 | - | - |
| **Item 85 - D** | *Shopping support* | Y | | No analysis. Variables are constants | | | | 52 |  |  |
| **Item 85 - E** | *Budgeting support* | Y | | Kappa | 1.00 | - | - | 52 | - | - |
| **Item 85 - F** | *Laundry support* | Y | | Kappa | 1.00 | - | - | 52 | - | - |
| **Item 86 - A** | *Psychoeducation - Multimedia* | Y | | Kappa | 0.95 | 0.87 | 1.00 | 51 | - | - |
| **Item 86 - B** | *Psychoeducation - Written information* | Y | | Kappa | 1.00 | - | - | 52 | - | - |
| **Item 86 - C** | *Psychoeducation - Patient support group* | Y | | Kappa | 1.00 | - | - | 52 | - | - |
| **Item 86 - D** | *Psychoeducation - Expert talks* | Y | | Kappa | 0.96 | 0.88 | 1.00 | 52 | - | - |
| **Item 86 - E** | *Psychoeducation provided by service* | New | | Kappa | 1.00 | - | - | 52 | - | - |
| **Item 87** | *Families invited to care meetings* | Y | | Kappa | 1.00 | - | - | 52 | - | - |
| **Item 88** | *Number of families currently involved* | N | | ICC | 1.00 | - | - | 48 | 0-94 | 11.98 |
| **Item 89** | *SUs with family meetings with staff* | Y | | ICC | 1.00 | - | - | 47 | 0-62 | 6.51 |
| **Item 90** | *Service users paid for work in facility* | N | | Kappa | 0.73 | 0.48 | 0.98 | 52 | - | - |
| **Item 91** | *Attending MH service training scheme* | Y | | ICC | 0.99 | 0.987 | 0.99 | 46 | 0-7 | 0.52 |
| **Item 92** | *Attending mainstream training scheme* | Y | | ICC | 1.00 | 1.00 | 1.00 | 46 | 0-20 | 0.80 |
| **Item 93** | *Attending social co-operative* | Y | | Scale has zero variance items | | | | 46 | 0-0 | 0.00 |
| **Item 94** | *Attending college course* | Y | | ICC | 1.000 | 0.999 | 1.000 | 46 | 0-40 | 2.67 |
| **Item 95 - A** | *Attending other course* | Y | | **ICC** | **0.650** | **0.444** | **0.792** | **44** | **0-1** | **0.05** |
| **Item 95 - B** | *Other course – Description* | N | | Descriptive item. No analysis. | | | |  |  |  |
| **Item 96** | *SUs work as volunteers in community* | Y | | ICC | 0.98 | 0.96 | 0.99 | 46 | 0-10 | 1.96 |
| **Item 97** | *SUs in paid employment last 12 months* | Y | | ICC | 1.00 | - | - | 46 | 0-4 | 0.46 |
| **Item 98** | *Have a vocational rehabilitation specialist* | N | | Kappa | 0.94 | 0.84 | 1.00 | 51 | - | - |
| **Item 99** | *Voc. rehab. specialists professional background* | N | | Descriptive item. No analysis. | | | |  |  |  |
| **Item 100** | *Hours per day SUs spend doing planned activity* | Y | | ICC | 1.00 | - | - | 29 | 1-12 | 3.20 |
| **Item 101** | *Number of SUs difficult to engage* | N | | ICC | 1.00 | - | - | 51 | 0-52 | 7.43 |
| **Item 102 - A** | *Links with local entertainment* | Y | | Kappa | 0.90 | 0.70 | 1.00 | 52 | - | - |
| **Item 102 - B** | *Links with cafes, restaurants* | Y | | Kappa | 1.00 | - | - | 52 | - | - |
| **Item 102 - C** | *Links with sports facilities* | Y | | Kappa | 1.00 | - | - | 52 | - | - |
| **Item 102 - D** | *Links with religious organisations* | Y | | Kappa | 1.00 | - | - | 52 | - | - |
| **Item 102 - E** | *Links with local neighbourhood organisations* | Y | | Kappa | 1.00 | - | - | 52 | - | - |
| **Item 102 - F** | *Regular activities in the unit* | Y | | Kappa | 1.00 | - | - | 52 | - | - |
| **Item 102 - G** | *Providing same programme for all* | Y | | Kappa | 0.86 | 0.71 | 1.00 | 52 | - | - |
| **Item 102 - H** | *Providing different programme for each* | Y | | Kappa | 0.95 | 0.86 | 1.00 | 50 | - | - |
| **Item 102 - I** | *Other* | Y | | Kappa | 1.00 | - | - | 52 | - | - |
| **Item 102 - J** | *Other – Description* | N | | Descriptive item. No analysis. | | | |  |  |  |
| **Item 103** | *Regularly take part in unit activities* | Y | | ICC | 1.00 | - | - | 39 | 0-23 | 7.56 |
| **Item 104** | *Regularly take part in activities in community* | Y | | ICC | 1.00 | - | - | 48 | 1-301 | 19.81 |
| **Item 105** | *Individual care plan* | Y | | No analysis. Variables are constants | | | | 52 |  |  |
| **Item 106 - A** | *SU involed* | Y | | Kappa | 1.00 | - | - | 52 | - | - |
| **Item 106 - B** | *Family member/carer involved* | Y | | Kappa | 1.00 | - | - | 51 | - | - |
| **Item 106 - C** | *Care co-ordinator involved* | Y | | Kappa | 1.00 | - | - | 52 | - | - |
| **Item 107** | *Identify patient priorities before care meeting* | Y | | Kappa | 1.00 | - | - | 50 | - | - |
| **Item 108** | *Patient present at care meeting* | Y | | Kappa | 1.00 | - | - | 50 | - | - |
| **Item 109** | *Care meeting multidisciplinary* | Y | | Kappa | 0.94 | 0.84 | 1.00 | 51 | - | - |
| **Item 110** | *Decisions negotiated with patient* | Y | | Kappa | 1.00 | - | - | 47 | - | - |
| **Item 111 - A** | *Decide waking time* | Y | | Kappa | 1.00 | - | - | 52 | - | - |
| **Item 111 - B** | *Decide sleeping time* | Y | | Kappa | 1.00 | - | - | 52 | - | - |
| **Item 111 - C** | *Decide how spend money* | Y | | Kappa | 1.00 | - | - | 52 | - | - |
| **Item 111 - D** | *Decide to spend time off unit* | Y | | Kappa | 1.00 | - | - | 50 | - | - |
| **Item 111 - E** | *Decide number of cigarettes* | Y | | Kappa | 1.00 | - | - | 52 | - | - |
| **Item 111 - F** | *Decide what alcohol to drink* | Y | | Kappa | 0.87 | 0.69 | 1.00 | 52 | - | - |
| **Item 111 - G** | *Decide who to date* | Y | | No analysis. Variables are constants | | | | 52 |  |  |
| **Item 111 - H** | *Decide to stay out overnight* | Y | | Kappa | 1.00 | - | - | 51 | - | - |
| **Item 112** | *Full control over finances* | Y | | Kappa | 1.00 | - | - | 52 | - | - |
| **Item 113** | *Personal budget* | New | | Kappa | 1.00 | - | - | 52 | - | - |
| **Item 114** | *Personal budget – Description* | New | | Descriptive item. No analysis. | | | |  |  |  |
| **Item 115** | *Consensual sex in unit* | Y | | Kappa | 1.00 | - | - | 49 | - | - |
| **Item 116** | *Support in manage own medication* | Y | | Kappa | 0.96 | 0.87 | 1.00 | 52 | - | - |
| **Item 117** | *Number who manage own medication* | N | | ICC | 1.00 | - | - | 50 | 0-100 | 9.98 |
| **Item 118** | *Number have non service user friends* | Y | | ICC | 1.00 | - | - | 32 | 0-105 | 9.56 |
| **Item 119** | *Advocacy available* | N | | Kappa | 1.00 | - | - | 52 | - | - |
| **Item 120** | *Number SUs that have used advocacy* | Y | | ICC | 1.00 | - | - | 44 | 0-42 | 3.57 |
| **Item 121** | *SUs on Section given info about civil rights* | Y | | Kappa | 1.00 | - | - | 41 | - | - |
| **Item 122** | *Info include access to lawyer* | Y | | Kappa | 1.00 | - | - | 35 | - | - |
| **Item 123** | *Info include access to interpreter* | Y | | Kappa | 1.00 | - | - | 34 | - | - |
| **Item 124** | *Welfare/benefits advice service* | Y | | Kappa | 1.00 | - | - | 52 | - | - |
| **Item 125** | *Percentage assisted to vote* | Y | | ICC | 1.00 | - | - | 52 | 0-100 | 92.50 |
| **Item 126** | *Staff have named supervisor* | Y | | No analysis. Variables are constants | | | | 52 |  |  |
| **Item 127** | *Frequency of supervision* | Y | | Kappa | 1.00 | - | - | 51 | - | - |
| **Item 128** | *Use group supervision* | Y | | Kappa | 1.00 | - | - | 52 | - | - |
| **Item 129** | *Frequency of group supervision* | Y | | Kappa | 0.92 | 0.75 | 1.00 | 20 | - | - |
| **Item 130** | *External review* | N | | Kappa | 1.00 | - | - | 52 | - | - |
| **Item 131** | *Months since last inspection* | N | | ICC | 0.99 | 0.98 | 0.99 | 47 | 0.5-48 | 10.41 |
| **Item 132** | *Inspecting organisation – Description* | New | | Descriptive item. No analysis. | | | |  |  |  |
| **Item 133** | *Records kept in locked environment* | Y | | No analysis. Variables are constants | | | | 52 |  |  |
| **Item 134** | *Formal complaints procedure* | Y | | No analysis. Variables are constants | | | | 52 |  |  |
| **Item 135** | *Policy for abuse, aggression, bullying from staff* | Y | | Kappa | 1.00 | - | - | 52 | - | - |
| **Item 136** | *Happy to have relative in unit* | N | | Kappa | 1.00 | - | - | 52 | - | - |
| **Item 137** | *Service location* | N | | Kappa | 1.00 | - | - | 33 | - | - |
| **Item 138** | *Entering unit* | N | | Kappa | 1.00 | - | - | 38 | - | - |
| **Item 139** | *Sign with name of unit* | N | | Kappa | 1.00 | - | - | 38 | - | - |
| **Item 140** | *Sign indicated mental health unit* | Y | | Kappa | 1.00 | - | - | 30 | - | - |
| **Item 141** | *External appearance* | Y | | **Kappa** | **0.69** | **0.52** | **0.87** | **37** |  |  |
| **Item 142** | *Internal appearance* | Y | | Kappa | 0.74 | 0.579 | 0.897 | 37 | - | - |
| **Item 143** | *Researcher happy to have relative in service* | N | | **Kappa** | **0.55** | **0.313** | **0.79** | **26** |  |  |
